# Anti-Prion Activity of Brilliant Blue G

**DOI:** 10.1371/journal.pone.0037896

**Published:** 2012-05-31

**Authors:** Yoshifumi Iwamaru, Takato Takenouchi, Yuichi Murayama, Hiroyuki Okada, Morikazu Imamura, Yoshihisa Shimizu, Makoto Hashimoto, Shirou Mohri, Takashi Yokoyama, Hiroshi Kitani

**Affiliations:** 1 Prion Disease Research Center, National Institute of Animal Health, Tsukuba, Ibaraki, Japan; 2 Animal Immune and Cell Biology Research Unit, National Institute of Agrobiological Sciences, Tsukuba, Ibaraki, Japan; 3 Division of Sensory and Motor Systems, Tokyo Metropolitan Institute of Medical Science, Setagaya-ku, Tokyo, Japan; The Scripps Research Institute Scripps Florida, United States of America

## Abstract

**Background:**

Prion diseases are fatal neurodegenerative disorders with no effective therapy currently available. Accumulating evidence has implicated over-activation of P2X7 ionotropic purinergic receptor (P2X7R) in the progression of neuronal loss in several neurodegenerative diseases. This has led to the speculation that simultaneous blockade of this receptor and prion replication can be an effective therapeutic strategy for prion diseases. We have focused on Brilliant Blue G (BBG), a well-known P2X7R antagonist, possessing a chemical structure expected to confer anti-prion activity and examined its inhibitory effect on the accumulation of pathogenic isoforms of prion protein (PrPres) in a cellular and a mouse model of prion disease in order to determine its therapeutic potential.

**Principal Findings:**

BBG prevented PrPres accumulation in infected MG20 microglial and N2a neural cells at 50% inhibitory concentrations of 14.6 and 3.2 µM, respectively. Administration of BBG *in vivo* also reduced PrPres accumulation in the brains of mice with prion disease. However, it did not appear to alleviate the disease progression compared to the vehicle-treated controls, implying a complex role of P2X7R on the neuronal degeneration in prion diseases.

**Significance:**

These results provide novel insights into the pathophysiology of prion diseases and have important implications for the treatment.

## Introduction

Prion diseases are fatal, transmissible, and progressive neurodegenerative disorders that include Creutzfeldt–Jakob disease (CJD) and Gerstmann–Straussler–Scheinker syndrome (GSS) in humans, bovine spongiform encephalopathy (BSE) in cattle, scrapie in sheep and goats, and chronic wasting disease in deer. The neuropathology is characterized by brain vacuolation, astrogliosis, microglial activation, neuronal loss, and progressive accumulation of a misfolded protease-resistant isoform (PrPres) of the host-encoded protease-sensitive prion protein (PrPsen) [1]. The conversion of PrPsen to PrPres is believed to be the key event in prion pathogenesis. Until date, the precise molecular mechanisms underlying glial activation and neuronal dysfunction remain unknown and there are no effective treatments for prion diseases.

P2X7 ionotropic purinergic receptor (P2X7R) is an ATP-gated ion channel believed to be associated with the regulation of both neuronal death and survival. P2X7R is abundantly expressed in microglia [2] and to a lesser extent in astrocytes [3], oligodendrocytes [4], and the presynaptic terminals of neurons [5]. Accumulating evidence suggests that the P2X7R signal pathways are involved in the modulation of glutamate release from presynaptic terminals of neurons and astrocytes [6]–[8], resulting in synapse dysfunction and glutamate-mediated excitotoxicity. Furthermore, signaling through P2X7R plays a crucial role in the activation and proliferation of microglia [9]. After activation of P2X7R by ATP, activated microglia release various proinflammatory cytokines (e.g., IL-1β) and other bioreactive substances, leading to neuronal damage [10]–[12]. In addition to its deleterious effects, P2X7R activation also stimulates the release of γ-aminobutyric acid from nerve terminals [6] and production of endocannabinoids in astrocytes and microglia [13], [14], both of which are transmitters with neuroprotective roles. P2X7R activation also involves a neuroprotective effect through activation of ERK1/2 signaling [15]. Thus, P2X7R activation can have both protective and detrimental effects on neurons.

P2X7R expression is upregulated in the brains of patients with and in various animal models of neurodegenerative diseases, including multiple sclerosis and Alzheimer's and Huntington's diseases [16]–[19]. In addition, we recently reported that P2X7R is upregulated in a mouse model of prion disease [20]. Although it remains debatable whether P2X7R plays a beneficial or detrimental role in these diseases, a number of studies illustrate that the blockade or deficit of P2X7R has neuroprotective effects in animal models of multiple sclerosis [4], Huntington's disease [19], Alzheimer's disease [21], and spinal cord injury [22]. While data regarding the role of P2X7R in prion diseases is lacking, the simultaneous blockade of this receptor and inhibition of prion replication may alleviate the progression of prion diseases.

One candidate for a therapeutic compound that possess such combined drug actions is Brilliant Blue G (BBG), a well-known P2X7R antagonist; BBG can cross the blood–brain barrier, has low toxicity, and exhibits therapeutic effects in several animal models of neurodegenerative diseases [23]. Furthermore, BBG has a symmetrical bifunctional structure comprising of two moieties joined via a spacer; this molecular framework is expected to confer anti-prion activities [24] as in the case of anti-prion compounds such as Congo red [25], suramin [26], and curcumin [27]. These properties prompted us to assay BBG for its ability to inhibit PrPres accumulation.

In this study, we examined the inhibitory effect of BBG on PrPres accumulation in a cellular and a mouse model of prion disease and we also investigated the therapeutic potentials of BBG due to its P2X7R antagonistic and predicted anti-prion activities. We found that BBG inhibited PrPres accumulation in prion-infected microglial and neural cell lines, possibly via downregulation of cell-surface PrPsen. BBG prevented PrPres accumulation in the brain of prion-infected mouse. However, BBG did not appear to alleviate the disease progression.

## Materials and Methods

### Reagents and antibodies

BBG (Ultra Pure Grade) was obtained from AnaSpec (San Jose, CA, USA) and all other reagents were purchased from (Sigma-Aldrich, St. Louis, MO, USA) unless otherwise specified. The following antibodies were used in this study: anti-prion protein (PrP) SAF32 monoclonal antibody (mAb) (SPI Bio, Montigny le Bretonneux, France); anti-PrP mAbs 3H2, 4E10, and T2 [28], [29]; anti-P2X7R rabbit polyclonal antibody (Sigma, P8232); anti-glial fibrillary acidic protein (GFAP) mAb (Sigma, G3893); anti-β-actin mAb (Sigma, A2228), anti-synaptophysin 1 mAb (Synaptic Systems, Gottingen, Germany); anti- ionized calcium binding adaptor molecule 1 (Iba1) rabbit polyclonal antibody (Wako Pure Chemical, Osaka, Japan); and HRP-conjugated goat anti-mouse and anti-rabbit antibodies (Calbiochem, San Diego, CA, USA).

### Cell culture conditions and inhibition assay of PrPres accumulation

MG20 cells persistently infected with mouse-adapted scrapie ME7 prion (ScMG20 cells) [30] were cultured in Dulbecco's modified Eagle's medium supplemented with 10% heat-inactivated fetal bovine serum, 10 µM 2-mercaptoethanol, 10 µg/ml insulin,100 units/ml penicillin, 100 µg/ml streptomycin sulfate at 37°C with 5% CO_2_. N2a cells were obtained from American Type Culture Collection. N2a cells persistently infected with mouse-adapted scrapie Chandler prion (ScN2a) were cultured in Opti-MEM supplemented with 10% heat-inactivated fetal calf serum at 37°C with 5% CO_2_.

The cells were detached by the addition of 5 mM EDTA in phosphate-buffered saline (PBS) with gentle pipetting, and cell density was determined by cell counting using an hemocytometer. ScMG20 and ScN2a cells were plated in 6-well plates (1×10^5^ cells/well) and allowed to settle for 1 h or 1day prior to the treatment with an antagonist, respectively. Cells were treated with BBG (0.6–60 µM) or without BBG, oxidized-ATP (25–150 µM; an irreversible inhibitor of P2X7R), or A438079 (4.5–36 µM; an inhibitor of P2X7R) for 3 days, after which the accumulation of PrPres was detected by western blotting of the lyzed cells.

### Cell viability

Cytotoxicity was determined using the WST-8 assay (Cell Counting Kit-8; Dojindo Lab, Kumamoto, Japan). ScMG20 cells were plated in the wells of a 96-well plate (3×10^3^ cells/well) and incubated with the appropriate concentration of one of the three antagonists for 3 days prior to the WST-8 assay, according to the manufacturer's instructions. The treatment with each substance was performed in triplicate. The percentage cell viability was calculated with reference to that of untreated cells incubated with WST-8 (100%).

### SDS-PAGE and western blotting

Cells were washed once with PBS and then lyzed in an ice-cold lysis buffer containing 10 mM Tris-HCl (pH 7.4), 150 mM NaCl, 5 mM EDTA with 0.5% Triton X-100, and 0.5% sodium deoxycholate. Frozen mice brain or mice spleen tissue was homogenized in an ice-cold lysis buffer containing 10 mM Tris-HCl (pH 7.4), 150 mM NaCl, 5 mM EDTA with 0.5% Triton X-100, 0.5% sodium deoxycholate, and 0.1% SDS. After removal of insoluble debris by centrifugation at 10,000× *g* for 2 min, the total protein concentrations of the samples were measured by the bicinchoninic acid assay (BCA protein assay; Thermo Fisher Scientific, Rockland, IL, USA) and equal amounts of protein were analyzed.

For PrPres detection, cell lysates and tissue homogenates were digested with proteinase K (20 and 50 µg/ml for the cell tissues and brain tissues, respectively) for 1 h at 37°C. The reactions were stopped by the addition of 4 mM Pefabloc (Roche Diagnostics, Basel, Switzerland). Cell lysates were then ultracentrifuged at 200,000× *g* for 1 h in a TLA 55 rotor (Beckman Coulter, Fullerton, CA, USA) and the resulting pellets were used for further analysis. The samples were solubilized in LDS-sample loading buffer (Invitrogen, San Diego, CA, USA) prior to electrophoresis on 12% NOVEX pre-cast gels (Invitrogen) and electrotransfered onto Durapore (Millipore, Billerica, MA, USA) polyvinylidene fluoride membranes. Chemilumi One Super (Nakarai Chemical, Kyoto, Japan) was used for immunodetection. For quantitation, blots were imaged with a Fluorchem (Alpha Innotech, San Leandro, CA, USA) and analyzed with Image Reader software (AlphaEaseFC; Alpha Innotech) according to the manufacturer's instructions. The values were normalized to β-actin as loading controls.

### Degycosylation analysis

For analysis of PrP levels, MG20 cells were incubated with the indicated concentrations of BBG. After 3 days, cell extracts were prepared in lysis buffer, and PrP was deglycosylated by treating the extracts with PNGase F (peptide N-glycosidase F) (New England BioLabs, Beverly, MA, USA) for 2 h at 37°C as described previously [30].

### Infectivity assay

The infectivity associated with ScMG20 cells was assayed by intracerebral inoculation into murine PrP-overexpressing (*tga*20) mice. ScMG20 cells were harvested for inoculation at serial passage 5 in the presence or absence of 30 µM BBG. The cells were suspended in sterile PBS(−), freeze-thawed, and sonicated before intracranial inoculation (2.5×10^4^ cells/20 µl/mouse). After inoculation, the terminally ill mice were sacrificed for western blotting of the brain tissues. All mice were kept in an air-conditioned room and fed on standard laboratory food pellets and water *ad libitum*.

### Detergent solubility assay

Detergent solubility assays were performed as previously described [26]. MG20 cells treated with or without 30 µM BBG were lyzed in cold lysis buffer. Postnuclear lysates were ultracentrifuged for 1 h at 100,000× *g* at 4°C in the presence of Complete proteinase inhibitor cocktail (Roche) and 1% N-lauryl sarcosine. PrPsen in the supernatant and pellet fractions was detected by western blotting.

### In vivo BBG treatment

C57BL/6 mice were intracerebrally infected with 20 µl of 10% homogenate prepared from the brains of either healthy C57BL/6 mice or terminally ill C57BL/6 mice with Fukuoka-1 (FK-1) strain of murine GSS. Hundred days after infection, mice were treated intraperitoneally 3 times per week for 3 weeks and 2 times per week for 4 additional weeks with 500 µl of 100 mg/kg BBG diluted with 10% ethanol in saline. A control group of GSS-infected animals was treated with the vehicle only.

### Confocal imaging

Cells grown on a 4-well chambered coverglass were incubated with BBG for 3 days. After rinsing with PBS, the cells were fixed with 4% paraformaldehyde in PBS for 30 min at room temperature and then treated with or without 0.5% Triton X-100 in PBS. After a brief wash with PBS, the cells were incubated with blocking solution (1% bovine serum albumin, 5% normal goat serum, and 0.1% Tween 20 in PBS) for 30 min. The primary antibody directed against PrP (SAF32) was used at a 1∶500 dilution in blocking solution and incubated for 1 h at room temperature. Cells were subsequently incubated with anti-mouse Alexa Fluor 488-conjugated secondary antibody (Invitrogen) for 1 h. The chambered coverglass was overlaid with a mounting solution [25% glycerol, 10% Mowiol (Calbiochem) in 100 mM Tris-HCl, pH 8.5] and examined with the Zeiss LSM510 inverse laser scan microscope.

### Flow cytometric analysis

Cells grown on 100-mm cell culture dishes were incubated with 30 µM BBG for 3 days. After rinsing with ice-cold PBS, cells were harvested by pipetting. Next, 2 µg of several anti-PrP antibodies were pre-labeled using the Zenon PE Mouse IgG Labeling Kit (Invitrogen), according to the manufacturer's instructions. Cells were incubated for 30 min at 4°C with each Zenon-labeled antibody and washed twice in PBS containing 1% BSA. Following fixation with 1% paraformaldehyde in PBS, cells were analyzed with the EPICS XL SYSTEM II flow cytometer (Beckman Coulter, Miami, FL). Fluorescence of 1.5×10^4^ cells/antibody was acquired and analyzed using Flowjo software (Tree Star, Ashland, OR, USA) to quantify the surface expression of PrP by calculating the mean fluorescence intensities.

### Protein misfolding cyclic amplification (PMCA) assay

Brains from healthy mice and Chandler scrapie-infected mice at the terminal stage of the disease were minced using the BioMasher (Nippi) and suspended in a conversion buffer. After removal of insoluble debris by centrifugation at 4,500× *g* for 5 min, the supernatants were collected. The Chandler brain homogenate was diluted 1∶1000, 1∶10000, and 1∶100000 with healthy brain homogenates. Serial dilutions of BBG in PBS were added to the brain homogenates to obtain final concentrations of 0.6, 6, 60, and 600 µM. The samples were incubated at 37°C with agitation for 30 min and then subjected to a cycle of sonication every hour, as previously described [31].

### Quantitative real-time reverse transcriptase polymerase chain reaction (Q-PCR) analysis

The mRNA levels were assayed by Q-PCR. Total RNA was isolated from cells with FastPure RNA Kits and treated on a column with RNase-free DNase I (Takara, Osaka, Japan). Total RNA was reverse transcribed using ReverTra Ace (Toyobo, Osaka, Japan) with random hexamers primers. Diluted reactions were analyzed with SYBR Premix Ex Taq (Takara) and an ABI Prism 7500 detection system (Applied Biosystems). PrP mRNA levels were normalized to those of glyceraldehyde-3-phosphate dehydrogenase (GAPDH). cDNA levels of PrP and GAPDH were determined relative to standard curves from PCR fragments amplified by RT-PCR using total RNA and a Q-PCR primer pair. The slope of the standard and dissociation curves of the products indicated sufficient PCR quality. The primers used were as follows: mouse PrP, (forward) 5′-ATGGCGAACCTTGGCTACTG-3′ and (reverse) 5′-CCTGAGGTGGGTAACGGTTG-3′; GAPDH, (forward) 5′-AGGTCGGTGTGAACGGATTTG-3′ and (reverse) 5′-TGTAGACCATGTAGTTGAGGTCAGATTTG-3′.

### Statistical analysis

Values in the figures are expressed as mean ± SD. To determine statistical significance, Welch's *t*-test for unpaired data was applied as appropriate. A value of *p*<0.05 was considered significant.

### Ethics Statement

Procedures involving animal subjects have been approved by the Institutional Animal Care and Use Committee at the National Institute of Animal Health (approval ID: 09-44 and 10-005).

## Results

### BBG inhibits PrPres accumulation and prion replication in mouse scrapie-infected cells

**Figure 1:**
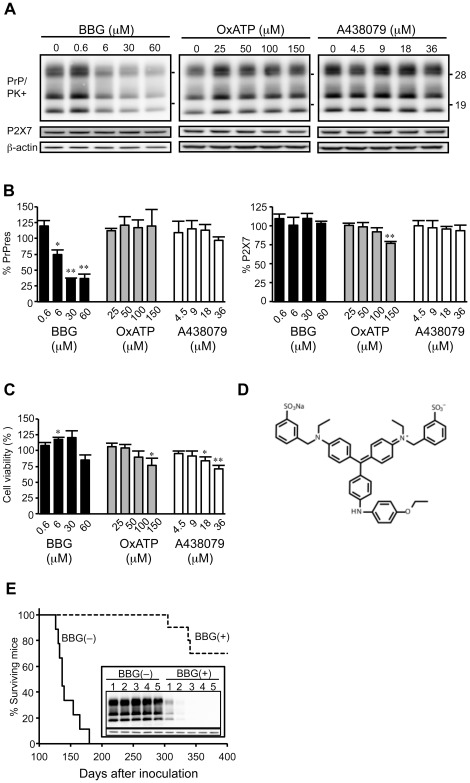
BBG prevents PrPres and prion accumulation in ScMG20 cells. (A) Western blotting of the levels of PrPres and P2X7 receptor proteins. ScMG20 cells were mock-treated or treated with each tested drug at the indicated concentrations for 3 days. PrPres and P2X7 receptor protein levels in ScMG20 cells were measured by western blotting. Molecular mass standards (kDa) are indicated on the right. Membranes were probed with anti-β-actin to correct any possible deviation in the protein concentration. (B) The densitometric measurement of PrPres and P2X7 receptor protein signals detected in the blots. Band intensity for PrPres and P2X7 receptor proteins was measured, normalized using the values obtained for b-actin intensities, and expressed as percentage relative to the 0 µM concentration points described in the Materials and Methods section. Results are expressed as the mean ± SD of three different experiments. Significant differences at a *p*<0.05 (^*^) and 0.01 (^**^) between treated and untreated cells are indicated. (C) The effects of P2X7 antagonists on viability of ScMG20 cells. Cell viability determined by the WST-8 assay is shown. The absorbance values are expressed as mean ± SD relative to the absorbance values of untreated control ScMG20 cells from 3 independent experiments. Significant differences at *p*<0.05 (^*^) and 0.01 (^**^) between treated and untreated cells are indicated. (D) Structures of Brilliant Blue G. (E) Kaplan–Meier survival analysis of *tga20* mice inoculated with ScMG20 cells treated with or without BBG. *tga20* mice were inoculated intracerebrally with ScMG20 cells in 5 serial passages in the presence or absence of 30 µM BBG. Animals that received BBG-treated cells (BBG+, broken line) showed longer survival times compared to those that received untreated cells (BBG−, solid line). The inset picture shows that the kinetics of PrPres levels in ScMG20 cells serially passaged 5 times in the presence or absence of 30 µM BBG. The numbers above the lanes indicate the passage number.

In the present study, we examined the effects of P2X7R antagonists on PrPres accumulation in cultured microglial cells persistently infected with the ME7 murine strain of scrapie (ScMG20 cells). Cells were treated with one of the three antagonists: BBG, oxidized-ATP, or A438079. BBG inhibited PrPres accumulation in a dose-dependent manner, with a 50% inhibitory concentration (IC50) of approximately 14.6 µM in ScMG20 cells (Figs. 1A, 1B). On the other hand, the levels of PrPres were not reduced following a treatment of cells with oxidized-ATP or A438079. The toxicity of the P2X7R antagonists was assessed using the WST-8 metabolic assay (Fig. 1C). ScMG20 cell viability decreased by approximately 20% at the highest concentration of each antagonist. However, BBG was not toxic at concentrations required for maximal inhibition of PrPres accumulation. These results suggest that anti-prion activity of BBG was derived from its molecular frameworks (Fig. 1D), which are analogous to the anti-prion compounds and not from its P2X7R antagonistic activity.

To determine the effects of BBG on prion replication, bioassays of ScMG20 cells treated with or without BBG were conducted. ScMG20 cells were serially passaged up to 5 times in the presence of 30 µM BBG. The level of PrPres in ScMG20 cells became undetectable after 3 passages with BBG (Fig. 1E insert). Inoculation of *tga20* mice (n = 10) with untreated ScMG20 cells resulted in a mean survival time of 151.5±27.8 days (mean ± SD). On the other hand, mice inoculated with BBG-treated cells showed a prolonged incubation period of the disease, with 6 out of the 10 inoculated mice not succumbing to the disease within 400 days after inoculation. We also examined the efficacy of anti-prion activity of BBG using neuronal derivative N2a cells infected with the Chandler murine strain of scrapie (ScN2a). BBG also prevented PrPres accumulation in ScN2a cells, with an IC50 of 3.2 µM (data not shown).

### BBG depletes cells of PrP and modifies its cellular localization

**Figure 2:**
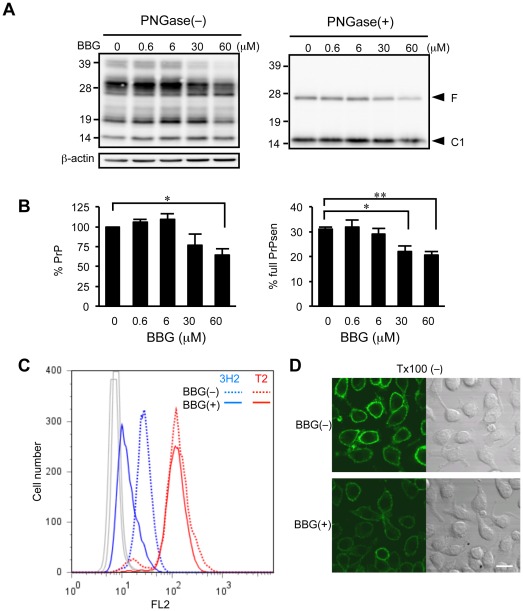
BBG depletes cells of PrPsen and mediates decrease in full-length PrPsen on cell surface. (A) Western blotting of the levels of PrPsen Uninfected MG20 cells were treated with or without 30 µM BBG for 3 days. PrPsen protein levels in MG20 cells were measured by western blotting. Molecular mass standards (kDa) are indicated on the left. The locations of full-length (F) and N-terminal truncated PrPsen (C1) are indicated. Membranes were probed with anti-β-actin to correct for any possible deviation on protein concentration. (B) The densitometric measurement of PrPsen signals detected in the blots after deglycosylation. The signals of PrPsen from BBG-treated cells are expressed as percentage of the signals from untreated cells (left panel). The signals of full-length PrPsen band intensities are expressed as a percentage of the signals from total PrPsen (full-length PrPsen plus C-terminal fragments) (right panel). Each bar indicates the mean values (±SD) of at least three independent experiments. Significant differences at *p*<0.05 (^*^) and *p*<0.01 (^**^) between BBG and untreated cells are indicated. (C) Fluorescence-activated cell sorting analysis of cell-surface expression levels of total PrPsen and full-length PrPsen of MG20 cells. The histograms depict the cell-surface expression in non-permeabilized cells incubated with 30 µM BBG for 3 days. FL2 represents the fluorescence intensity in BBG-treated cells (solid line) and in the untreated cells (dashed line), plotted against the number of cells (events). For each cell population, 10,000 events were measured. Mabs 3H2 was used for detection of full-length PrPsen (blue line), and Mabs T2 was used for detection of the total PrPsen (red line) on the cell surface. (D) Confocal fluorescence microscopic analysis of full-length PrPsen on the cell surface of MG20 cells. Cells were incubated with or without 30 µM BBG for 3 days. MG20 cells growing on cover glasses were fixed with 4% paraformaldehyde. Confocal fluorescent images (left panels) and Nomarski differential interference contrast images (right panels) were obtained. Scale bar, 20 µm.

The level of endogenous PrPsen influences the rate of replication of prions [32] and a reduction in its level abrogates PrPres formation [33]. To examine whether the reduction of PrPres by BBG was mediated by the suppression of PrPsen, the PrPsen level in uninfected MG20 cells treated for 3 days with various concentrations of BBG was ascertained. Cell lysates not treated with proteinase K (PK) were subjected to western blotting, and the total PrPsen (full-length PrPsen plus C-terminal fragments) levels were determined after the removal of carbohydrates by PNGaseF (Fig. 2A). At a concentration of 60 µM, BBG significantly reduced the total PrP levels and mediated a reduction in the ratio of full-length PrPsen to total PrPsen, suggesting a preferential decrease in full-length PrPsen (Fig. 2B).

To determine whether BBG affected PrPsen synthesis, we examined the expression levels of PrPsen mRNA in MG20 cells treated with or without 30 µM BBG by Q-PCR. The PrPsen expression level was calculated as the ratio of the number of PrPsen copies to the number of GAPDH copies. The mean expression level of PrPsen in BBG-treated cells was 0.028±0.001 (mean ± SD of 3 independent experiments), which was slightly lower than that of 0.031±0.001 in untreated cells. This result suggests that the reduction of PrPsen might be, at least in part, associated with the downregulation of its expression.

PrPsen cycles between the cell surface and the endosomal compartments, and PrPres formation is postulated to occur in this pathway [34]–[38]. To determine whether BBG treatment can influence PrPsen localization, the cell-surface expression of PrPsen was investigated by fluorescence-activating cell sorting (FACS) analysis. Mabs 3H2, which reacts with linear amino acid epitope 38–53, was used to detect full-length PrPsen and Mabs T2, which reacts with the discontinuous epitope spanning amino acids 132–156, was used to assess the total PrPsen. BBG-treated cells showed a decrease in the level of full-length PrPsen levels on the cell surface as compared with untreated cells (Fig. 2C); this result was consistent with the western blotting results.

The effect of BBG on the cellular localization of PrPsen was also examined by confocal microscopy. To detect only full-length PrPsen, we used SAF32, which reacts with linear amino acid epitopes 59–89. In mock-treated non-permeabilized cells, full-length PrPsen was found to be localized at the cell surface. In the presence of BBG, a marked decrease of full-length PrPsen at the cell surface was detected (Fig. 2D). These results demonstrate that BBG interrupts the cell-surface expression of PrPsen.

### Misfolded PrPsen is undetectable in BBG-treated cells

Previous studies have demonstrated that several anti-prion compounds cause misfolding of PrPsen, as evidenced by the relative detergent insolubility and formation of intracellular aggregates, resulting in the downregulation of surface PrPsen by altering its distribution inside the cell or inducing its rapid degradation (e.g., dextran sulfate, pentosan polysulfate, and epigallo catechin gallate) [26], [39], [40]. Thus, it is possible that the reduction in full-length PrPsen levels at the surface of BBG-treated cells is due to the misfolding of PrP.

**Figure 3:**
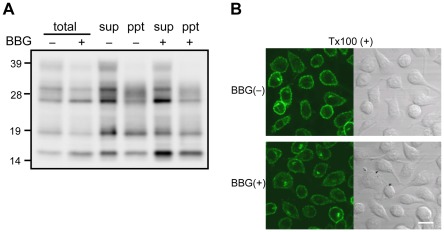
BBG does not induce formation of the detergent-insoluble PrPsen and the intracellular aggregated PrPsen. (A) Insoluble PrPsen detection in MG20 cells by detergent solubility assay. After incubation with or without 30 µM BBG for 3 days, cells were lyzed and postnuclear lysates were adjusted to 1% N-lauryl sarcosyl and ultracentrifuged for 1 h at 100,000× *g*. PrPsen present in the detergent-soluble (sup) and detergent-insoluble (ppt) fractions was analyzed by western blotting using monoclonal antibody T2. (B) Confocal fluorescence microscopic analysis of permeabilized MG20cells. Cells were incubated with or without 30 µM BBG for 3 days. MG20 cells growing on cover glasses were fixed with 4% paraformaldehyde and incubated with 0.1% Triton X-100. Confocal fluorescent images (left panels) and Nomarski differential interference contrast images (right panels) were obtained. Scale bar, 20 µm.

To investigate this possibility, MG20 cells were treated with or without 30 µM BBG followed by a detergent solubility assay. Cells were lyzed in a detergent buffer, and the presence of PrPsen in the detergent-soluble and detergent-insoluble fractions was analyzed by western blotting. PrPsen was detected in both the detergent-soluble and, to some extent, the detergent-insoluble fractions (Fig. 3A). There was no significant difference in the level of detergent insoluble PrPsen in the presence or absence of BBG. Furthermore, the cellular localization of PrPsen in BBG-treated cells was visualized by indirect immunofluorescence of permeablized cells. Similarly, there was no observable difference in the intracellular localization of PrPsen in presence or absence of BBG (Fig. 3B).

### BBG does not affect PrPres amplification in vitro

**Figure 4:**
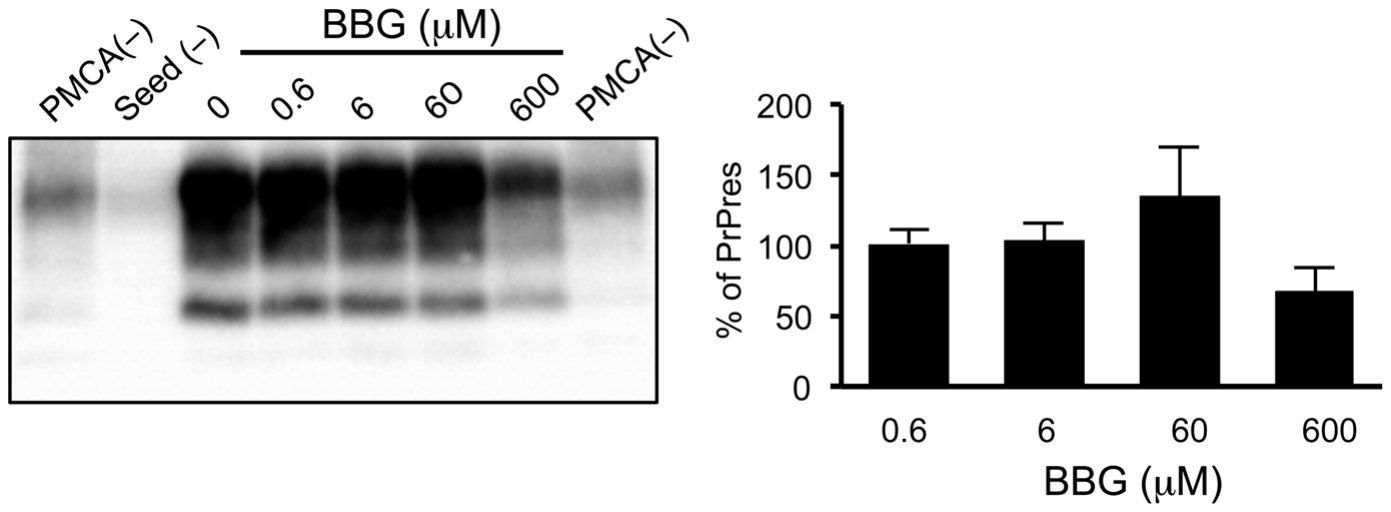
PrPres amplification in the presence of BBG by PMCA. (A) Western blotting of PrPres amplified by PMCA. The brain homogenates from mice with Chandler scrapie were added to normal mice brain homogenates to prepare final dilutions of 1∶1000. PMCA was performed with BBG at indicated concentrations. After PK digestion, PrPres was detected by immunoblot with Mab T2. (0) represents samples without BBG, PMCA(−) represents samples without amplification and seed(−) represents samples for which PMCA was performed but without seed inoculumn. (B) Densitometric analysis of PK-resistant PrP signals in panel A. The signals of PrPres band intensities are expressed as a percentage of the PrPres signals from non-treated (0) cells. Each bar indicates the mean values (±SD) of at three independent experiments.

To assess the possibility of the direct influence of BBG on PrPres formation, we utilized the PMCA technique [41]. PMCA was performed with chandler-infected mouse brain homogenate as the seed in the presence of BBG. For evaluating the amplification of PrPres after the PMCA reaction, the PK-resistant bands were semi-quantified by densitometric analysis. The presence of BBG in the PMCA reaction buffer did not significantly affect the amplification of PrPres except at an extremely high concentration (600 µM) (Fig. 4). These results suggest that BBG exert its effect on PrPres accumulation via complex cellular processes.

### BBG reduces the cerebral accumulation of PrPres but does not prolong the survival time of murine GSS-infected mice

**Figure 5:**
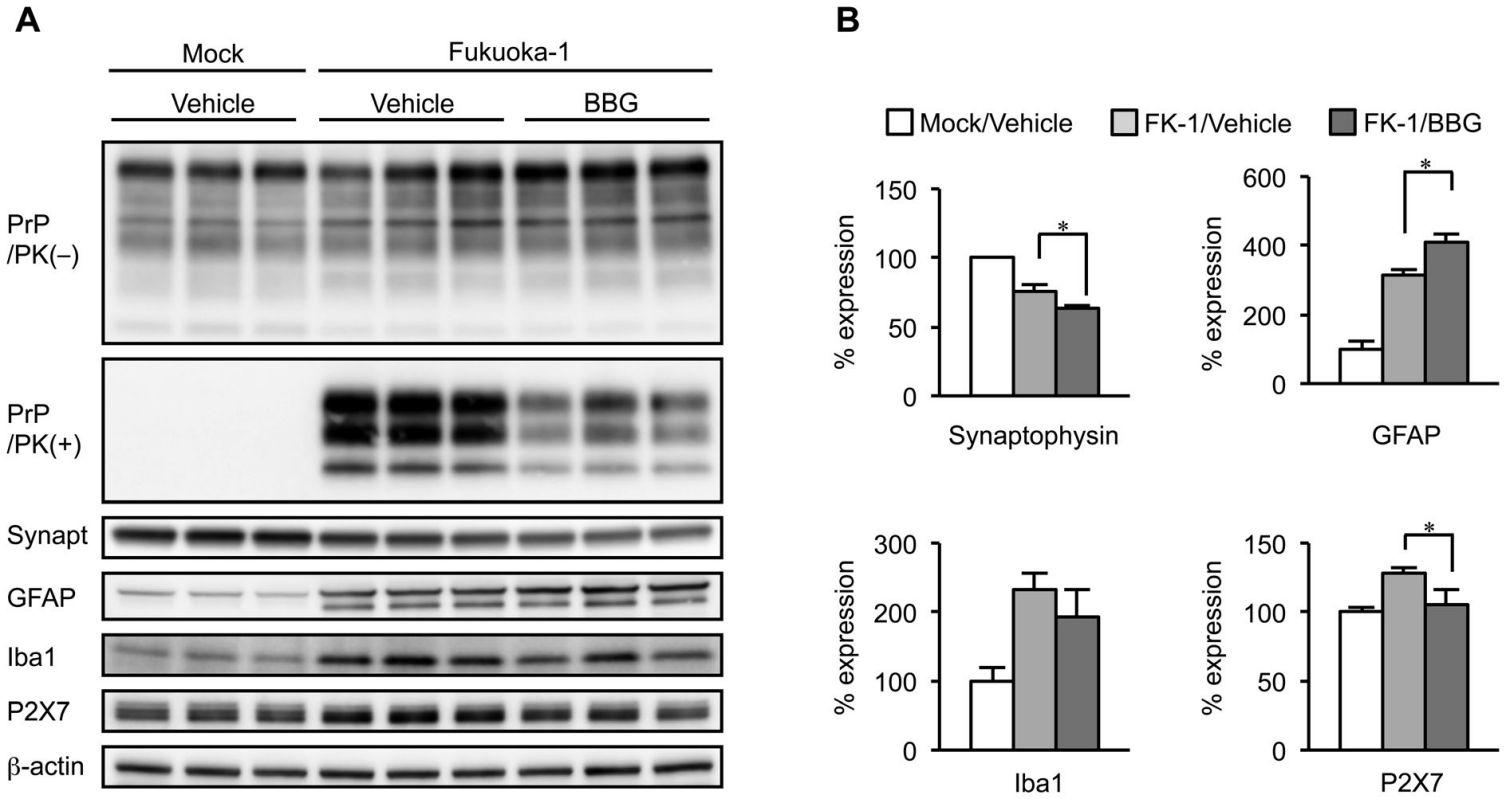
BBG prevents cerebral PrPres formation but not prevents disease progression. (A) Western blotting performed on the brain homogenates of Fukuoka-1 (FK-1) strain of murine GSS infected C57/BL6 mice treated with 100 mg/kg BBG for 3 times per week intraperitoneally for 3 weeks. The control mock and FK-1 infected animals were treated with the vehicle only. Membranes were probed with antibodies against indicated antigens. (B) Densitometric analysis of marker proteins in panel A. Each bar represents the average ratio from triplicate samples with mock-infected mice as standard. Significant differences at a *p*<0.05 (^*^) between the BBG- and vehicle-treated animals are indicated.

Lastly, we tested whether BBG could affect prion disease and prevent the disease progression during the later stages of infection. Hundred days after intracerebral inoculation with a high dose of FK-1 murine GSS strain (at 60% disease duration), mice were intraperitoneally treated with 100 mg/kg BBG 3 times per week for 3 weeks. In order to assess the effect of BBG on the pathological brain changes, some of the treated mice (n = 3) were euthanized and their brain homogenates were subjected to semi-quantitative western blotting at 121 days after infection (Fig. 5A, 5B). The remaining mice (n = 7) were continuously treated with 100 mg/kg BBG 2 times per week for a total of 7 weeks. In the brains of the mice treated with BBG for 3 weeks, the PrPres signal obtained using mAb T2 was reduced by 43% compared with that in the vehicle-treated control animals (p<0.01).

It has been shown that in murine prion disease, impaired synaptic protein, synaptic loss, and gliosis occur at an early stage [42]–[46]. Thus, the extent of synaptic loss and gliosis usually reflects the severity of the prion disease. Consistent with these reports, western blotting of the brain homogenates from mice infected with FK-1 strain demonstrated that synaptophysin levels were decreased by approximately 30% and GFAP and Iba1 levels were elevated by approximately 310% and 230%, respectively, compared with those in mock-treated control mice. In accordance with a previous report [20], P2X7R levels were elevated by approximately 30% in diseased mice.

Surprisingly, in mice infected with FK-1 and treated with BBG, synaptic loss (synaptophysin marker) and astrogliosis (GFAP marker) were enhanced by 20% and 30%, respectively, despite the apparent reduction in the levels of PrPres. Microgliosis (Iba1 marker) tended to decrease, however the reduction was not significant. P2X7R levels were decreased by approximately 20%. As a BBG-treatment control, we examined levels of PrP, GFAP, Iba1 and synaptophysin in the brains of mock-infected mice treated with vehicle or BBG (100 mg/kg, 3 times per week for 3 weeks). No statistically significant differences in these protein levels between vehicle and BBG treated animals were observed, suggesting no detectable detrimental effect of BBG in healthy animals at this dosage (data not shown).

In the infected mice treated with BBG for 7 weeks, no significant delay in disease progression was observed (Table 1). In addition, the level of PrPres did not differ significantly between the BBG- and vehicle-treated mice at the end of the study (data not shown). These results suggest that BBG administration slows down PrPres accumulation in the brains, but has no therapeutic efficacy in a mouse model of GSS during the later stages of infection.

**Table 1:** Effect of intraperitoneally administered BBG on animals cerebrally inoculated with Fukuoka-1 GSS strain.

Treatment	Mice (diseased/inoculated)	Mean incubation time (days ± SD)
Vehicle	7/7	161.9±5.5
BBG (100 mg/kg) starting at 100 dpi	7/7	163.3±5.0

dpi: days post inoculation.

## Discussion

We showed here that BBG, a P2X7R antagonist, prevented PrPres accumulation in prion-infected MG20 microglial (IC50 14.6 µM) and N2a neural (IC50 3.2 µM) cell lines. *In vivo* administration of BBG, which was predicted to exert both anti-prion and P2X7R antagonistic activities, reduced PrPres accumulation in the brains of mice with prion disease, but it did not appear to alleviate the disease progression.

Among the three P2X7R antagonists examined, only BBG inhibited PrPres accumulation in a dose-dependent manner and reduced prion infectivity in cultured cells. These results suggest that its anti-prion activity was derived from its molecular frameworks, which are analogous to the well-known anti-prion compounds, such as Congo red, suramin, and curcumin, and not from its P2X7R antagonistic activity.

Several mechanisms can account for the effect of BBG on prion replication. We propose that BBG mediated the removal of PrPsen from the plasma membrane and reduced the substrate for PrPres formation within the endocytic pathway. Numerous studies have shown that perturbation of PrPsen cellular trafficking results in inhibition of PrPres formation, indicating that PrPres formation happens either on the cell surface or along the endocytic pathway [34]–[38], [47]. The manner in which BBG reduces the cell-surface PrPsen remains unclear. One possibility is that BBG might influence PrPsen synthesis. In uninfected MG20 cells, endogenous PrPsen levels were significantly reduced after incubation with BBG. In addition, there was a slight downregulation of PrPsen at the mRNA levels, which might explain the suppression of the cell-surface PrPsen.

Alternatively, BBG may directly interact with PrPsen on the cell surface or with other cellular components that influence PrPsen trafficking and accelerate the endocytosis of the cell-surface PrPsen. Several sulfated glycans decrease the amount of the cell-surface PrPsen by enhancing its rate of endocytosis through an unidentified mechanism associated with their binding to the N-terminal region of PrP, which is rich in basic amino acids and contains a consensus site for heparin and other sulfated glycans [40]. In addition, the conformational change or oligomerization of PrPsen at the cell surface induced by epigallocatechin gallate and copper lead to downregulation of cell-surface PrPsen [39], [48]. Although a direct interaction between BBG and PrP remains to be confirmed, it is conceivable that the binding of BBG to the polybasic N-terminal of PrPsen could lead to its internalization since the interaction of BBG with proteins is known to be mediated by basic and aromatic amino acids [49]. On the other hand, a BBG-induced conformational change in PrPsen is unlikely since we did not detect structural alterations of PrPsen in the presence of BBG, which are characterized by the relative detergent insolubility and formation of intracellular aggregates.

The third possibility regarding the BBG-mediated reduction of cell-surface PrPsen is the enhanced degradation of PrPsen. The reduction of PrPsen levels can be mediated by induced lysosomal, proteasomal, and autophagic degradations of PrPsen under certain conditions [26], [50], [51]; there were not evaluated in this study. Whether BBG accelerates the degradation of PrPsen remains to be determined.

The anti-prion activity of BBG did not appear to relate to a destabilization of pre-existing PrPres aggregates. Incubation of the cell lysates with BBG before PK treatment did not reduce PrPres band intensity (data not shown). In addition, BBG did not significantly modify PrPres in the brain homogenates in a PMCA reaction. This observation excludes the possibility that BBG modifies the western blotting detection of PrPres due to an artifact.

We hypothesized that BBG would display therapeutic efficacy against prion diseases *in vivo* based on its anti-prion activity and the neuroprotective effects of pharmacological antagonism of P2X7R reported in several animal models of neurodegenerative diseases. Unexpectedly, we demonstrated that BBG did not alleviate the progression of prion diseases. Two possibilities may explain the lack of neuroprotection following BBG administration, which was intended to pharmacologically antagonize P2X7R. It is most likely that activation of P2X7R plays an important role in neuroprotective effects under pathological conditions. Activation of P2X7R increases the production of endocannabinoids in microglial cells and astrocytes [13], [14]. Sustained production of endocannabinoids reduces the release of glutamate and cytotoxic agents from neuron and microglia, respectively, and increases the release of growth factors, leading to neuroprotection [52]–[54]. Indeed, activation of cannabinoid receptors by cannabinoid derivatives mediates neuroprotection in animal models of prion disease [55]. Thus, it is conceivable that BBG might inhibit the neuroprotection pathways involving P2X7R by antagonizing this receptor.

A second possible explanation for the lack of a protective effect of BBG is that the effects of BBG may be attenuated by suppressed microglial inflammatory responses in prion diseases. It is widely accepted that over-activated microglia release potentially toxic products, including reactive oxygen species, excitotoxin, and proinflammatory cytokines such as interleukin (IL)-1β, IL-6, and tumor necrosis factor-α, and the P2X7R system is essential for the induction of microglial activation [9]. In the case of animal models of prion disease, despite marked astrocytosis and microglial activation, typical proinflammatory cytokine expression is absent or low at the protein level [56]–[58] compared with those induced by lipopolysaccharide. It has been proposed that such a suppressed inflammatory response is mediated by the downregulation of microglial responses by an antiinflammatory cytokine, such as transforming growth factor-β1 [59]. In addition, prion induced-glial cytokine responses are mainly mediated by astrocytes [56], [57]. Taken together, we hypothesize that under such atypical inflammatory conditions characterized by restrained microglial response, BBG cannot exert neuroprotective effects because these effects have been attributed, at least in part, to its antiinflammatory activity of blocking microglial P2X7R [21]. This hypothesis is supported by previous results using an animal model of Parkinson's disease, where an atypical inflammatory response was observed [60] and pharmacological blockade of P2X7R by BBG and genetic deletion of this receptor had no protective effect on dopaminergic neurons [61]. Whether atypical inflammatory responses occurred in our mouse model of GSS remains to be determined. In addition, more research will be required to assess the correlation between the neuroprotective efficacy and antiinflammatory ability of BBG.

The change of brain pathology following BBG administration did not appear to relate to its potential toxicity of BBG. BBG is a derivative of a commonly used blue food dye (FD&C blue No. 1) and had been shown to be safe in healthy animals [62], [63]. Furthermore, we preliminary compared levels of PrP, GFAP, Iba1 and synaptophysin between brains of mock-infected mice treated with vehiclle or BBG (100 mg/kg, 3 times per week for 3 weeks). No statistically significant differences in these protein levels between vehicle and BBG treated animals were observed (data not shown). These previous and our observations exclude the possibility that the potential toxicity of BBG influenced brain pathology in prion-diseased animals.

In summary, BBG prevented PrPres accumulation in both a cell culture and a mouse model of prion disease. Although BBG possessed anti-prion and highly selective P2X7R antagonistic activities, it did not appear to ameliorate the disease progression. This suggests that the observed P2X7R upregulation in the brains of diseased mice may play a neuroprotective role in prion disease. These results provide insight into the pathophysiology of prion diseases and have important implications for the treatment of these disorders. Despite the lack of a neuroprotective effect, BBG has many therapeutic advantages, including anti-prion activity, low toxicity, and permeability across blood–brain barrier. Therefore, continued study of BBG as a potential anti-prion compound is warranted, taking into consideration its antagonistic effect of P2X7R. Combining BBG and cannabinoids may prove effective in the treatment of prion-infected animals.
